# Catalytic Cracking As the Basis for a Potential Detector For Gas Chromatography

**DOI:** 10.6028/jres.092.024

**Published:** 1987-08-01

**Authors:** Thomas J. Bruno

**Affiliations:** National Bureau of Standards, Boulder, CO 80303

**Keywords:** catalytic cracking, detector, gas chromatography

## Abstract

This short paper describes the design, construction and preliminary experimental results obtained with a potential new detector for the gas chromatographic analysis of hydrocarbon species. The functional principle of the detector is the measurement of the temperature change of a catalyst as catalytic cracking occurs on its surface. The catalyst is a silicon dioxide-aluminum oxide-zeolite mixture similar to the materials used commercially in industrial riser crackers. The temperature drop which occurs at the onset of cracking is measured using two opposed thermocouple junctions. The first prototype, described in this paper, consists of a single pair of junctions. After appropriate signal conditioning (using a commercially available filter-amplifier), the thermocouple output is logged using an electronic integrator. Work on multi-junction cells, which is currently in progress, is also described briefly.

## Introduction

The detection of the separated components from a gas chromatographic column is usually based upon the measurement of some property of the analyte species which is easily and clearly distinguishable from that of the carrier gas [1,2].^1^ Included among the many detectors developed for gas chromatography have been several that are based upon the analyte undergoing a catalyzed reaction [3–8]. In most of these approaches, catalysis results in the production of reaction products to which conventional gas chromatographic detectors are more sensitive, or in the production of an ion current which is measured using a very sensitive electrometer. Thus, in most of these detectors, the chemistry occurring during the catalyzed reaction and the nature of the catalyst itself are of secondary importance. This short note is a report of preliminary work on a potential detector which senses the temperature change of a cracking catalyst during reactions with appropriate hydrocarbon species.

Catalytic cracking has been of immense industrial importance since its introduction in 1927 [9]. The development of the technology of catalytic cracking has resulted in 10 commercial processes available for license. Because of the pressures of the marketplace, much of the science behind this mature technology remains buried in the vaults of proprietary secrecy. The catalytic cracking process is known to proceed through a carbonium ion mechanism [10]. This is to be contrasted with thermal cracking, which occurs via a free radical mechanism. Catalytic cracking is an endothermic process which requires a net input of heat to be applied to the system [11]. The major products of the catalytic cracking of normal hydrocarbons (up to n-hexadecane) are C_3_ to C_6_ branched hydrocarbons. Cracking catalysts have virtually no effect on low molecular weight hydrocarbons such as propane or n-butane. The ease of cracking usually increases with the hydrocarbon molecular weight. Among aliphatic hydrocarbons, cracking is accelerated by tertiary groupings, but is retarded by quarternary groupings (for example, that found in 2,2-dimethylbutane). Thus, a molecule may possess functionalities that have competing effects toward the cracking process [12].

Catalysts for cracking are usually formulated from a silica-alumina mixture which contains 10 to 20 percent aluminum oxide. Recently, the addition of up to 15 percent synthetic sodium alumino-silicate (type X zeolite molecular sieve) has provided greater catalytic activity. Commercial catalysts often have trace quantities of rare earth metals or platinum, but this is mainly done to assist in the process of hydrocracking (cracking followed by hydrogenation).

## Experimental

**Figure 1:**
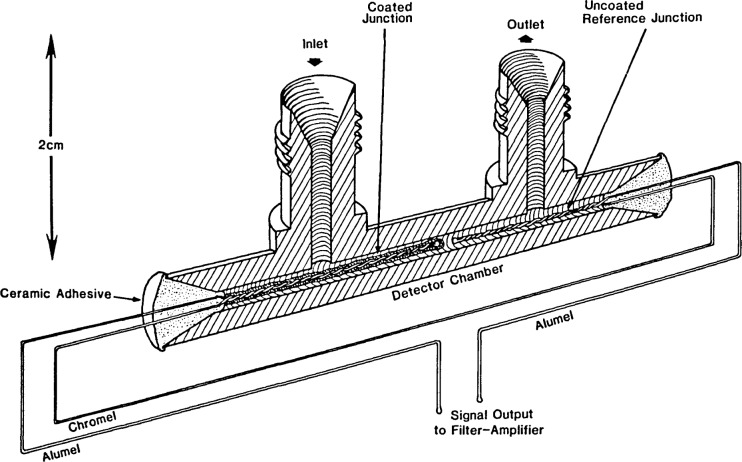
Catalytic cracker detector having the reference junction in the gas stream.

**Figure 2:**
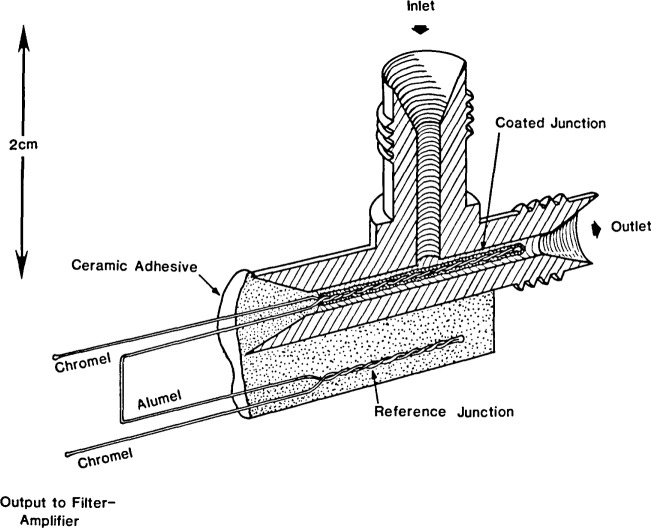
Catalytic cracker detector having the reference junction mounted remotely from the gas stream.

The two modifications of the prototype catalytic cracker detector which have been tested to demonstrate the potential of the approach are depicted in figures 1 and 2. The detectors are similar in concept to the adsorption detectors which have been designed for liquid chromatography [13] and supercritical fluid chromatography [14]. Each detector consists of two K-type (chromel-alumel) thermocouple junctions. One of the junctions is coated with catalyst, while the other serves as a reference. The catalyst employed was a mixture of 70 percent silicon dioxide, 20 percent aluminum oxide, and 10 percent sodium alumino-silicate (13-X type molecular sieve). The source of silicon dioxide was a high thermal conductivity silica based ceramic adhesive which contains a small percentage of binders. A thin layer of the mixture (suspended in water) was coated on the twisted strands of each sensing junction. The junctions were then mounted within the detector chamber (made from 316 stainless steel) using a zirconia-based gas-tight ceramic adhesive. In the first modification (fig. 1), the reference junction is mounted directly inside of the detector chamber, downstream from the sensing junction. In the second modification, the reference junction is mounted external to the detector chamber, potted in the zirconia ceramic adhesive mentioned earlier. In each case, the assembled detector cell is mounted inside of a copper block (to integrate out temperature variations) which is then placed inside of a small muffle furnace. The muffle furnace is positioned next to the column outlet of a commercial gas chromatograph. The inlet port of the detector is preceded by approximately 25 cm of stainless steel tubing (0.16 cm o.d., 0.03 cm i.d., not shown in the figures). This length of tubing serves as a heat exchanger (inside the muffle furnace) to preheat the carrier gas and analyte before they enter the detector cell. This heat exchanger is approximately 10 times the length necessary to provide 99 percent temperature equilibration. In subsequent modifications of this detector, the heat exchanger length will be optimized, and the transfer line length (from the column) will be minimized. The detector cell and heat exchanger tube are maintained at 550 °C during operation. This is the temperature at which catalytic cracking is optimum in commercial riser crackers using the silica-alumina- zeolite catalyst mixture.

As mentioned earlier, the functioning of the prototype catalytic cracker detector is based upon the measurement of the decrease in temperature of the coated junction (upon contact with a crackable hydrocarbon) with respect to the uncoated (fig. 1) or remote (fig. 2) reference junctions. Type K thermocouples provide a potential difference of 43 *μ*V/deg in the temperature range of interest [17]. The output of the thermocouples is fed into a commercial filter-amplifier designed for chromatographic applications. The output of this device is then sent on to a commercial electronic integrator.

The filter-amplifier was used with a gain setting of 1, and only served to decrease short term noise. It is, in fact, possible to eliminate this device altogether and feed the detector signal directly to the integrator. Chromatograms which are quite acceptable may be obtained in this fashion.

## Preliminary Results

**Figure 3:**
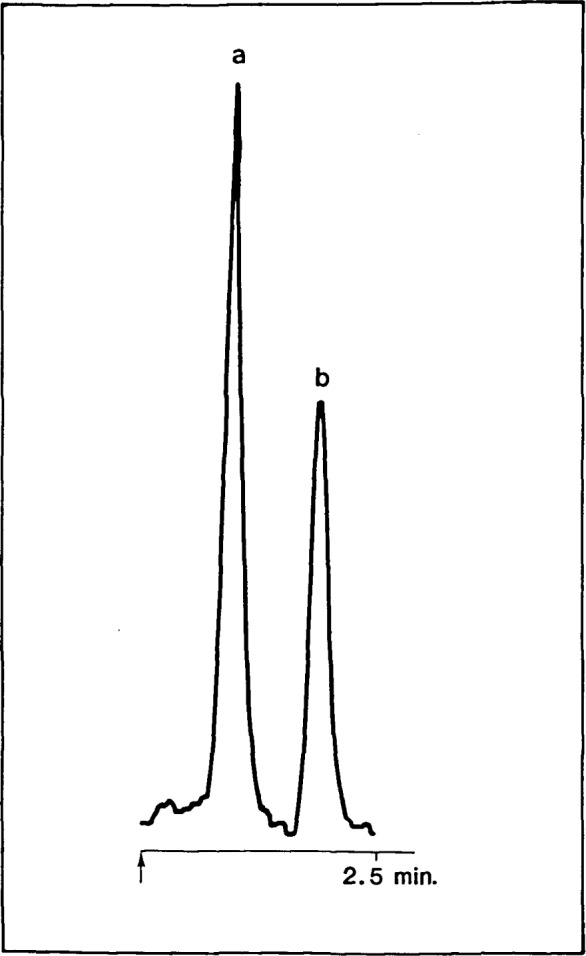
Chromatogram showing the response of the detector to n-hexane (a) and n-octane (b).

A sample chromatogram showing the detector response to 5*μ*1 of a mixture of n-hexane and n-octane (60/40 approximate mole percent) is shown in figure 3. This chromatogram was obtained using the detector depicted in figure 1. The separation was performed on a 3 m packed column of Porapak-QS [1],^2^ at a column temperature of 200 °C, using helium (at a flow-rate of 30 mL/min) as the carrier gas. The integrator-recorder was programmed for a full-scale sensitivity of 8 *μ*V. Thus, the peaks obtained correspond to a catalyst temperature drop of between 0.12 °C and 0.16 °C. The response of the detector is selective for crackable hydrocarbons. Thus, samples of n-butane and propane were unresponsive, as was a mixture of freons. The detector responds also to alkyl substituted aromatic hydrocarbons, with the response increasing with the size of alkyl substituent. This observation is expected in view of the work by Haensel [10]. Benzene gives an erratic response, since at elevated temperatures (and in contact with stainless steels) the preferred reaction is the formation of biphenyl [18,19].

After several weeks of operation, a deposit of coke was found to have formed upon the surface of the coated junctions. This is expected, since coke formation always occurs in commercial riser crackers. The coke layer did not cause a noticeable decrease in the activity of the detector, probably because of the relatively small quantity of hydrocarbon undergoing reaction in the detector relative to that commercial situations. The coke layer is easily burned off in the presence of air.

## Conclusions

The preliminary work on the catalytic cracker detector shows that the device has a good deal of potential as the basis of a detector for the selective analysis of hydrocarbons. It does not require a continuous stream of carrier gas, as does a thermal conductivity detector (TCD), nor does it require the fuel and oxidant of a flame ionization detector (FID). The circuitry required for the detector is extremely simple, consisting of a low-cost filter amplifier instead of the bridge circuit (for the TCD) or the electrometer (for the FID). These features make the catalytic cracker detector attractive for continuous line process analysis in an industrial environment, where safety, durability and simplicity is of paramount concern.

In its present form, the prototype detector has some major drawbacks. The first and most important is the low sensitivity (approximately three orders of magnitude lower than the TCD). We are addressing this problem by constructing multiple- junction thermopile cells in order to boost the signal level. Results with a six-junction cell have been very encouraging, with a nearly five-fold increase in sensitivity being observed. The six-junction prototype is similar in concept to that shown in figure 1. It is clear that very significant increases in sensitivity are possible with multiple junction cells. We are currently constructing a 12-junction thermopile cell which incorporates a flow-through catalyst design. Measurement of sensitivity and linear dynamic range, as well as testing the effects of different carrier gases and flow-rates, will be performed on this model. Current work on the multijunction cells is done using a commercially available proportional SCR temperature controller instead of the muffle furnace. This device provides far better temperature control and uses only 10 percent as much power as the muffle furnace.

## Footnotes

1 Figures in brackets indicate literature references.

2 Certain commercial equipment, instruments, or materials are identified in this paper in order to adequately specify the experimental procedure. Such identification does not imply recommendation or endorsement by the National Bureau of Standards, nor does it imply that the materials or equipment identified are necessarily the best available for the purpose.
